# Self-care behaviors related to air pollution protection questionnaire: a psychometric analysis

**DOI:** 10.1186/s13690-020-00400-7

**Published:** 2020-04-10

**Authors:** Mehdi Mirzaei-Alavijeh, Farzad Jalilian, Elena-Niculina Dragoi, Razieh Pirouzeh, Laleh Solaimanizadeh, Shima Khashij

**Affiliations:** 1 Social Development & Health Promotion Research Center, Health Institute, Kermanshah University of Medical Sciences, Kermanshah, Iran; 2 Faculty of Chemical Engineering and Environmental Protection “Cristo for Simionescu”, “Gheorghe Asachi” Technical University, BldMangeron no 73, 700050 Iasi, Romania; 3 Department of Nursing, Faculty of Nursing and Midwifery, Bam University of Medical Sciences, Bam, Iran

**Keywords:** Environmental health, Health education, Preventive behavior, Health promotion

## Abstract

**Background:**

Air pollution is an environmental health issue that has received much attention due to its harmful effects on public health. The aim of this study was to determine the psychometric properties of the self-care behaviors related to air pollution protection. To this mean, questionnaire responses provided by Iranian college students were analyzed and a Health Belief Model (HBM) determined.

**Methods:**

The self-care behavior related to air pollution protection was determined from responses from 301 university students using test item characteristics, face validity, reliability (internal consistency) and construct validity. Moreover, we carried out an exploratory factor analysis (EFA) to determine the factorial structure and explained variance.

**Results:**

Based on Eigenvalues of ≥1.00 and factor loadings of ≥0.40, seven factors were extracted. The calculated Kaiser–Meyer–Olkin value was 0.804. Overall, the seven factors explain 66.39% of the variance in the hypothesized model. Cronbach’s alpha for the measured factors: the perceived susceptibility, severity, barriers, benefits, self-efficacy, cues to action and behavior were 0.85, 0.79, 0.86, 0.66, 0.74, 0.83, and 0.75, respectively.

**Conclusion:**

The analysis of the questionnaire’s reliability and validity properties resulted in good values. The questionnaire is a promising instrument to assess self-care behavior related to air pollution protection from the perspective of college students.

## Background

Due to the fact that it causes diseases (such as respiratory, cardiovascular and cancers), the exposure to air pollution has been associated with a decrease of life expectancy and quality of life and with an increase in mortality and morbidity [1–5]. The United Nations Environment Program (UNEP) estimated that, worldwide, approximately 1.1 billion people breathe unhealthy air (approximately 80% of the world population [6, 7] and that the urban air pollution annually is responsible for 4.6 million lost years of healthy life [8]. Therefore, designing and developing preventive programs to reduce the exposure to air pollution is of stringent urgency [9].

The most important task in the development of preventive programs is the identification of effective determinants [10], various studies focused in evaluating the behavior of individuals at different ecological levels [11–14]. Given the complexity of human behavior, psychologists and social scientists have recommended the use of theories and models to identify and explain the fine aspects of human behavior [15]. The focus of health psychology studies is on the factors that influence how people adopt and change health behaviors [16]. It was found that the main factors that influence health behaviors are based on cognitive factors such as beliefs, attitudes and processes used when an individual makes decision [15]. One of the most widely used theories to explain the health behaviors of individuals is the Health Belief Model (HBM) [16]. HBM, as a psychological model, predicts health behaviors by focusing on an individual’s cognitive constructs, attitudes, and beliefs. The key determinants of HBM are: perceived susceptibility, severity, benefits, barriers, self-efficacy, and cues to action [17]. The HBM has also been successfully used to predict and develop health intervention strategies [18, 19]. After the cognizance of the behaviors and related cognitive determinants, one of the most important tools needed for health program planners is designing and developing standard questionnaires to evaluate them. Next, the psychometric evaluation, the most important step in standardizing the developed questionnaires to measure the behaviors and related cognitive determinants [20] is applied. Considering the lack of studies in developing countries, our study focused on development and psychometric properties of the air pollution self-care behavior questionnaire in a sample of Iranian college students.

## Materials and methods

### Participants

This cross-sectional study was conducted in 2017among301 male and female university students in Kermanshah University of Medical Sciences (KUMS) the west of Iran and 12 experts.

#### Questionnaire development

For the questionnaire development, a pool of items based on HBM constructs mentioned by Rosenstock et al. (perceived susceptibility, perceived severity, perceived benefit, perceived barrier, perceived self-efficacy, and cues to action) and behaviors [17] about self-care behavior related to air pollution protection was used in addition with mechanisms from similar studies [18, 19, 21–24] and in accordance with expert panel comments. The expert panel included two health policymakers, four environmental health specialists, one health services manager, and five health educators and promoters. Items were carefully written to minimize ambiguity and increase comprehension. In total, the item pool was comprised of 29 items. A five-point Likert type scaling was used for responders, ranging from 1 (strongly disagree) to 5 (strongly agree).

#### Psychometric

##### Face validity evaluation

Face validity indicates that the measured elements are apparently capable of measuring the concept of the research. In other words, face validity represents to what extent the items are similar to the subject in terms of appearance, rationality, proportionality, attractiveness, logical sequence of items, utility and importance of the instrument, with an emphasis on the target group’s perspective [25]. The face validity of the self-care behavior related to air pollution protection questionnaire was evaluated qualitatively. Thus, face-to-face individual interviews were held up with 12 experts, their comments analyzed and the necessary modification performed.

##### Content validity evaluation

Content validity can be defined as the ability of selected items to reflect the characteristics of the construct. Content validity is a way of making sure that the items used are capable of measuring the concept [26]. The self-care behavior related to air pollution protection questionnaire content validity was measured by both quantitative and qualitative methods. In this case, similar to the face validity evaluation, 12 experts were interviewed and their comments about the difficulty, relevance, and the ambiguity were examined, and the items modified based on their comments. In addition, in order to measure quantitative content validity, 12 other experts were interviewed to assess whether each item was “essential”, “useful but not essential”, or “not essential”. The expert comments were used to measure the Content Validity Ratio (CVR) and Content Validity Index (CVI). The CVR is used to ensure that the most important and correct content (item necessity) is selected and CVI is used to ensure that the tool items are optimally designed to measure the content. Based on the Lawshe table, the minimum value for acceptable CVI and CVR items were considered 0.79 and 0.62, respectively [27, 28].

##### Construct validity evaluation

The construct validity indicates how well a test is up to its claims; this type of validity refers to whether the operational definition of a variable actually reflects the true theoretical meaning of a construct [29]. Construct validity was investigated through the use of Classical Item Analysis (CIA) and Exploratory Factor Analysis (EFA). For this purpose, university students in Kermanshah University of Medical Sciences (KUMS) voluntarily agreed to participate in the study. Evidence shows that five or ten subjects per item are required for factor analysis, while some researchers suggest three samples per item of instrument [30]. In the present study, we considered approximately, eleven subjects for every item of instrument.

KUMS has 4742 students in 7 faculties including (a) dentistry, (b) medical, (c) pharmacy, (d) public health, (e) nutrition and food sciences, (f) nursing & midwifery, and (g) allied medical sciences. To select the participants, a three-step sampling technique (cluster sampling, proportional and randomization) was used. First, each school was considered as a cluster and all seven clusters of KUMS were included in the study. Then, the sample size for each school (cluster) was determined based on its population coverage (the number of students studying there). Finally, due to high numbers of students in each school, the participants for the study were selected randomly using Excel randomization. In total, 319 students from all 7 clusters were selected. From this population, 301 (94.3%) voluntarily agreed to participate in the study and signed the consent form. After that, the volunteers received the self-questionnaire. Finally, the data collected was used to evaluate classical item analysis and exploratory factor analysis. Item analysis was evaluated by using the Corrected Item-Total Correlation (CITC), and items with a CITC less than 0.4 were excluded from the final questionnaire; CITC refers to the correlation between each item and a scale score that excludes that item [31]. To perform exploratory factor analysis, the Kaiser-Meyer-Olkin (KMO) test and Bartlett’s test of sphericity were used to evaluate of sampling adequacy.

The KMO specifies that factor analysis is possible on the data stored. It examines the intensity of correlations between variables or questions; it should be noted that the acceptable value of KMO for a factor analysis is 0.7 [29]. One of the main assumptions in factor analysis is there must be a correlation between variables examined through the use of Bartlett Test of Sphericity [31].

Factor assumptions and analysis is should be as simple as possible; each factor must be based on factor analysis and evaluated using Bartlett Test of Sphericity [31]. Moreover, EFA with VARIMAX rotation (a cutoff point of 0.4 for factor load) was used to determine the main factors of the inventory. VARIMAX rotation is used to simplify the expression of a particular sub-space in terms of just a few major items [29]. The determined factors were approved by using the scree plot. The scree plot selects the appropriate number of factors in EFA from the Eigenvalues and shows the Eigenvalues on the y-axis and the number of factors on the x-axis. In addition, the cut-offs Eigenvalues of ≥1.00 and factor loadings of ≥0.40 were considered for selected factors [27]. Students in the KUMS represent the inclusion criteria, while lack of interest to participate and incomplete questionnaires were considered as excluded criteria.

##### Reliability evaluation

The internal consistency was measured by using Cronbach’s Coefficient Alpha of the various components of the HBM. All the data analysis was performed using the statistical package for social sciences (IBM SPSS) (Version 16.0).

### Research ethics

The research ethics committee at the deputy of research of KUMS approved the study protocol and monitored the research process (KUMS.REC.1396.438). Furthermore, students had been given adequate information about the purpose of the study. The individual personal information was kept confidentially.

## Results

The mean age of participants was 22.35 years [95% CI, 22.12, 22.59], ranged from 19 to 30 years. 52.8%of them were female and approximately 54% were BSc students. About 75.4% of participants were living in student dormitory. 29.9 and 29.6% of the respondents reported that their father had high school and academic education, respectively. In addition, 37.9% of the respondents reported that their mother had only primary school education. 52.2% of the students had average economic status. More details of demographic characteristics of the participants are shown in Table 1.

**Table 1:** Demographic characteristics of the participants

Variables	Number	Percent
Sex		
Female	159	52.8
Male	142	47.7
School		
Medicine	70	23.3
Dental	29	9.6
Pharmacology	41	13.6
Health	29	9.7
Nutrition and Food Sciences	12	3.9
Allied Medical Sciences	73	24.3
Nursing and Midwifery	47	15.6
Living in dormitory		
Yes	227	75.4
No	74	24.6
Fathers Education Level		
Primary school (grades 1–6)	68	22.6
Secondary school (grades 7–9)	54	17.9
High school (grades 10–12)	90	29.9
Academic (grades 13–16)	89	29.6
Mothers Education Level		
Primary school (grades 1–6)	114	37.9
Secondary school (grades 7–9)	64	21.3
High school (grades 10–12)	79	26.2
Academic (grades 13–16)	44	14.6
^a^Family Economic Status (Describing someone’s equipment’s such as owning a house, furniture, car, etc)		
Weak	22	7.3
Average	157	52.2
Good	109	36.2
Very Good	13	4.3

^a^ Participants were asked to select one of the following phrases to describe their family economic status: ‘Weak’, ‘Average’, ‘Good’ or ‘Very good’

None of the original twenty-nine items were omitted in the content validity process. The feedback from the panel experts was used to make minor editorial changes. There were three items with less than 0.40 CITC, which were omitted (Table 2).

**Table 2:** Items deleted in CIA

No	Item	Construct	Reason of item deleted
1	It is unpleasant to me if I use a mask when leaving home.	Perceived Barrier	CITC under 0.4
2	Using the face mask is unpleasant.	Perceived Barrier	CITC under 0.4
3	I believe that I can do air pollution self-care behaviors.	Perceived Self-efficacy	CITC under 0.4

**Fig. 1:**
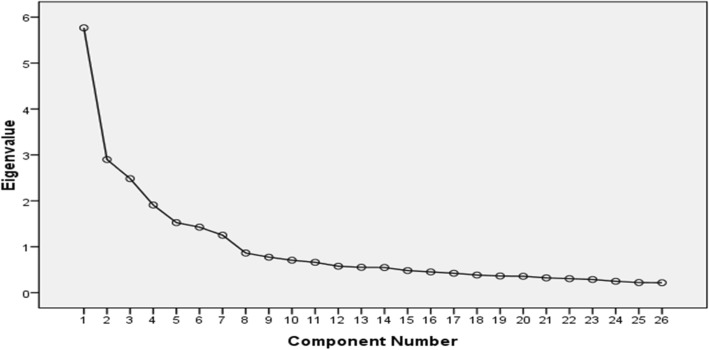
The scree plot of the factors studied among the participants

In addition, the twenty-six finalized items were applied for the explanatory and confirmatory factor analysis. The KMO test, which is the efficiency index of the sampling, was measured at 0.804. Bartlett’s Test was also significant (*P* < 0.001) which indicated the data is appropriate for factorial analysis. Based on Eigen values of ≥1.00 and factor loadings of ≥0.40, seven factors explain 66.39% of the variance. Furthermore, as it can be seen in Table 3, the estimates of the reliability using Cronbach’s coefficient alpha and the reliability coefficient for the all HBM variables suggested that the internal consistency was adequate. More details of exploratory factor analysis are shown in Table 3.It should be noted that the factor loadings of less than 0.4 are not shown in Table 3. Furthermore, the scree plot diagram of factors is shown in Fig. 1, where the slope of the curve becomes emergent at the seventh point.

**Table 3:** Obtained result of the exploratory factor analysis^*^

No	Items	)1(Perceived Severity	)2(Cues to Action	)3(Perceived Benefit	)4(Perceived Susceptibility	)5(Perceived Self-efficacy	)6(Perceived Barrier	)7(Behavior
(4) Perceived Susceptibility								
1	If I don’t use of face mask, during air pollution, I wouldn’t have any bad side effects.				0.786			
2	I would have chances for serious health consequences as a result of air pollution.				0.819			
3	In my age, the probability of air pollution complications is very low.				0.801			
(1) Perceived Severity								
1	Air pollution is a serious health problem.	0.605						
2	Complications due to air pollution may negatively affect my mental health.	0.799						
3	Complications due to air pollution may increase my educational problems.	0.749						
4	Air pollution can increase the cost of healthcare.	0.786						
5	Air pollution may lead to cancer.	0.733						
(6) Perceived Barrier								
1	My body is strong; air pollution does not effect on me.						0.581	
2	Air pollution is not a serious problem in my life.						0.703	
3	Using the face mask attracts attention.						0.749	
4	Using the face mask is costly.						0.701	
(3) Perceived Benefit								
	Air pollution prevention behaviors …							
1	Causes peace of mind.			0.783				
2	Decreases the need to refer to health care centers.			0.844				
3	Improves health.			0.825				
4	Reduces health costs.			0.779				
(5) Perceived Self-efficacy								
1	I am confident that, outdoors, I can use face mask respirators.					0.803		
2	I am confident that I can stay indoors in emergency cases of air pollution.					0.830		
3	I am confident that I can stay indoors at peak hours of air pollution.					0.749		
(2) Cues to Action								
1	How many of your friends are using the face mask in order to prevent of air pollution side-effects.		0.813					
2	Health care workers encourage me to the use of face mask.		0.798					
3	If I use face mask, my family will confirm it.		0.831					
4	If I use face mask, my friends will confirm it.		0.817					
(7) Behavior								
1	Staying indoors in emergency times of air pollution.							0.833
2	Staying indoors in the peak hours of traffic emission.							0.760
3	Using face mask respirators outdoors.							0.716
–	Variance (%)	22.17	11.14	9.55	7.34	5.86	5.49	4.81
–	Total Variance	66.39						
–	Cronbach’s Alpha Values	0.79	0.83	0.66	0.85	0.74	0.86	0.75

* Factors loadings of less than 0.4 are not shown in the Table 3

## Discussion

Our finding indicated that the internal consistency reliability of each component was equal to 0.66 to 0.86, which suggested that the items within each component of the HBM contributed to the precision of measurement. Overall, the current study indicated that the seven factors explained 66.39% of the variance in the hypothesized model. These findings showed that the questionnaire has acceptable validity and reliability and can be used in studies to assess Iranian students’ beliefs about air pollution prevention behavior. Several studies have been conducted to assess the validity and reliability of environmental health related scale whose methodological approach has been similar to our study [32–34]. For example, in Australia, Higginbotham et al. examined the validity and reliability of the environmental distress scale and reported that their scale construct (that includes hazard perception, felt impact changes, loss of solace, threat appraisal and environmental action) has strong (0.79–0.96) internal consistency reliability [32]. In line with our result, Berberoglu and Tosunoglu, in their study among 192 Turkish students stated that Environmental Attitude Scale (EAS) is a reliable and valid measure [33]. Deguen et al. carried out research on 2522 subjects in eight French cities with the aim of assessing the psychometric properties of air quality perception scale and indicated that their scale has good validity and reliability and can be used in studies for investigation of community perceptions of air quality [34]. Fernández-Manzanal et al. showed need to increase environmental education among university students [35]. It should also be acknowledged that educational campuses (such as universities) are unique in terms of access to opportunities for social and cultural advancement [36]. Hence, the availability of standard and native scales can be very useful for assessment and design of interventions.

Our findings indicated that the perceived severity, cues to action, perceived benefit, perceived susceptibility, perceived self-efficacy, perceived barrier, and behavior explained 22.17%, 11.14, 9.55, 7.34, 5.86, 5.49, and 4.81% of the variance in the hypothesized model, respectively. The perceived severity refers to individual feelings on the seriousness of getting an illness or engages in an unhealthy behavior [17]. Several studies have reported that perceived severity can play an important role in explaining health-related behaviors. For example, Claeson et al. indicated that perceived health risk perception was the best predictor of environmentally induced annoyance [21]. The results of a systematic review indicated the perceived severity of air pollution is one of the cognitive determinants of adherence to health advice recommendations of air quality warning systems [22]. In addition, cues to action are the stimulus needed to produce the decision-making to uptake a suggested behavior [17]. Cordano et al. focused on Chile and the United States students and noted that the norms determinant had the strongest association with pro-environmental behavior intention [23]. This is similar to the cues to action investigated in the current study. The perceived benefits were introduced as the third determinant, which played a significant role in estimating the variance of the model. These findings are similar to ones reported by previous researches. In this regard, Claeson et al. suggested if an individual believes that exposure to the material is hazardous, it could be a positive motivation to avoid exposure [21]. Radisic et al. indicated that the perceived benefits of healthy air quality had a significant relationship with air pollution exposure behavior [18, 19]. Actually, cognitive determinants as a theoretical framework for desirable behaviors are used for different types of behaviors. Consequently; a greater focus on social-cognitive determinants of the self-care behaviors related to air pollution protection is an effective and convenient strategy to develop successful preventive interventions.

The first important limitation of this study is that it did not assess the external validation of scale with the goal of establishing relationships to similar scales. The second limitation is that the data collection was just among sample of medical university students in the west of Iran; thus, this result cannot be generalized to other population groups. Finally, the current data collection is based on self-reporting, which often faces the risk of recall bias.

## Conclusion

This study offers a useful scale to measure self-care behavior related to air pollution protection and help in the development and planning prevention programs for air pollution for Iranian students. The analysis of the scale’s reliability and validity properties resulted in good values. The scale is a promising instrument to assess self-care behaviors related to air pollution protection from the perspective of university students. This scale could be used by health promotion planners to develop and implement air pollution protection promotion programs.

## Data Availability

Please contact the corresponding author for data requests.

## Abbreviations

BSc: Bachelor of Science
CIA: Classical Item Analysis
CITC: Corrected Item-Total Correlation
CVI: Content Validity Index
CVR: Content Validity Ratio
EAS: Environmental Attitude Scale
EFA: Exploratory Factor Analysis
HBM: Health Belief Model
KMO: Kaiser–Meyer–Olkin
KUMS: Kermanshah University of Medical Sciences
SD: Standard Deviation
SPSS: Statistical Package for Social Sciences
WHO: World Health Organization
